# The Effect of Neonicotinoid Insecticide and Fungicide on Sugar Responsiveness and Orientation Behavior of Honey Bee (*Apis mellifera*) in Semi-Field Conditions

**DOI:** 10.3390/insects9040130

**Published:** 2018-09-29

**Authors:** Xingchuan Jiang, Zhengwei Wang, Qibao He, Qiongqiong Liu, Xinyang Li, Linsheng Yu, Haiqun Cao

**Affiliations:** 1 College of Plant Protection, Anhui Agricultural University, Hefei 230036, China; jxc678@sina.cn (X.J.); heqibao0418@126.com (Q.H.); ahbb1104@163.com (Q.L.); lixydl@163.com (X.L.); 2 Chemical Ecology Group, CAS Key laboratory of Tropical Forest Ecology, Xishuangbanna Tropical Botanical Garden, Chinese Academy of Sciences, Kunming 650000, China; wangzhengwei@xtbg.ac.cn; 3 College of Animal Science and Technology, Anhui Agricultural University, Hefei 230036, China; yulinsheng@ahau.edu.cn

**Keywords:** carbendazol, oilseed rape, insecticide development, post-consumption syndrome, thiamethoxam

## Abstract

Neonicotinoid insecticides are in widespread use around the world, cause pollinator decline. We used semi-field conditions to determine the effect of sublethal insecticide, thiamethoxam, exposure on orientation behavior and sugar responsiveness. Bees could not reject the non-treated flower or the insecticide or insecticide/fungicide treated flower. After bees consumed the insecticide or insecticide/fungicide treated nectar, they could not discriminate between a flower odor or blank control in a Y-maze when making a first choice. We also found that treated bees wander back and forth in both arms to make a final decision about food location, and used longer duration in the Y maze than the control group. Sugar responsiveness was also reduced after bees were fed with insecticide or insecticide/fungicide treated food, one week was needed for them to display the same level of responsiveness as the control group. The thiamethoxam or thiamethoxam/carbendazol treated crop field does not act as an olfactory repellent to the bee, but it does affect its post-consumption behavior.

## 1. Introduction

Global declines in honeybees have been reported to be driven by agrochemicals, pathogens, climate change, and habitat loss [1,2]. A growing body of evidence shows neonicotinoid insecticides may act as a key factor in global pollinator decline [3,4].

Insecticides are designed to control specific pests in agro-ecosystem. However, these insecticides may also have an effect on non-target organisms. Non-target effects of insecticides first drew attention to humans, birds, and some endangered feral mammals [5]. Recent research however has also reported that insecticides are linked to insectivorous birds [6], butterflies [7] and wild bee decline [8,9]. Since pollinators such as bees play a crucial function in ecosystem services and agricultural crop yields, the decline of global pollinators has begun to call for additional legislation or prohibition of the use of neonicotinoid insecticides [1].

Neonicotinoids are very toxic to bees, cause pollinator decline [10] and have been shown to increase mortality in honeybees by reducing foraging success and impairing their homing ability [11]. Even though not all insecticides affect bee larval developmental rate, or survival, but still some of the insecticides, such as chlorpyrifos, did [12]. A worldwide survey of neonicotinoids in honey confirmed that there is an exposure risk of neonicotinoids in honey throughout the world, while the concentration was around 1.8 ng which was below the maximum residue level for human consumption [3].

In practice, farmers adopt neonicotinoid insecticide (thiamethoxam, THX) to control the pests, such as beetles (*Phyllotreta vittata*) and aphids (*Myzus persicae*, *Lipaohis erysimi*, and *Brevicoryne brassicae*), and fungicide (carbendazol, CAR) are used to reduce the harmful effects of the fungal infection (*Sclerotinia* stem rot). This common neonicotinoid insecticide, thiamethoxam, has been reported to impair the honey bee flight ability [13] and to alter bee motor functions and phototaxis [14]. This combinatorial exposure to insecticides would increase the propensity of a bee colony to collapse [15]. In a field survey near a corn growing region, bees would encounter a neonicotinoid/fungicide mixture, which consequentially renders them a high risk of reduced fitness [16]. According to degradations of different agrochemicals, the insecticide residue from the crop field may contaminate the surface water and soil close by, which in turn influence nearby beekeeping operations [17]. The plants close to the crop field would also be contaminated [18]. These insecticides and fungicides could enhance the side effects on non-target animals which feed on food/water in the crop field, even on the margins of the crop field with a synthesis of effects. However, the field surveys on the synthetic effects of multiple insecticides, or of a insecticide/fungicide mixture in close proximity to the crop field are variable [18]. This makes it difficult to evaluate the risk of agrochemicals to non-target animals like bees.

Caged experiments determined that the sublethal concentration (LC_50_) of thiamethoxam was 4.28 ng a.i. μL^−1^ on honeybees [19]. While sulethal doses (0.75 mg/g a.i.) of carbendazol do not cause bee acute death, but restrain the bee larvae growth and development [20]. However, in the field, the concentration of thiamethoxam and carbendazol are difficult to determine, because the bees may forage on alternative flowers to dilute the contaminated nectar, or even stimulate a detoxification process in the colony. To try and bridge the gap of knowledge between laboratory experiments and field work in determining thiamethoxam effects on bees, we need not only to take into consideration the concentration effects, but also timing effects.

Farmers often use a combination of multiple insecticides or insecticides/fungicide mixtures to treat different pests and diseases. The neonicotinoid insecticide (thiamethoxam, THX) and fungicide (carbendazol, CAR) were both commonly used in rape fields to control both pest and fungal infection. However, the side effect of these chemicals on non-target insects (i.e., the honey bee *Apis mellifera ligustica*) remains unknown in semi-field conditions. To better understand how honey bee react to agrochemicals used in the field, and when bees may recover from such an exposure, this study tested the orientation behavior and sucrose responsiveness of honeybees after chemicals were applied in a semi-field condition (netted greenhouses) two weeks after exposure. 

## 2. Materials and Methods

### 2.1. Agrochemicals

THX (30% suspension concentrate, SC) and CAR (50% wettable powder, WP) were purchased from the Insecticide Factory of Institute of Plant Protection, Chinese Academy of Agricultural Sciences (Beijing, China), and Jiangsu Sanshan Pesticide Co., Ltd., (Jiangsu, China), respectively.

### 2.2. Insect Treatments

Three colonies of the Western honey bee (*Apis mellifera ligustica*) were introduced into each nylon net covered greenhouse (size: 10 m × 15 m, height: 3.5 m) during rape (*Brassica napus* L.) flowering season three days before chemicals were used. Chemicals were applied once in each greenhouse. Bee colonies were moved out during a chemical treatment. The second morning the bee colonies were introduced back to the same place in the greenhouse. Bees could forage on rape flowers freely during experiments.

Three identical greenhouses were used in our experiments, one was treated with THX, one was treated with a mixture of THX and CAR, and the last one was treated with water only as a control. These two agrochemicals were diluted with ddH_2_O, and the recommended doses (90 g a.i. hm^−2^ for THX and 1290 g a.i. hm^−2^ for CAR) were used in this experiment to spray on rape flowers. All the treatments were treated with a sprayer in order to mimic the practical applications that farmers actually apply to rape flowers. Sunshine and wind could easily go through the greenhouse, but bees could not fly out of the greenhouse to forage on the other flowers. Bees from each colony were collected on the 1st, 3rd, 5th, 8th, 11th and 14th day to test the orientation behavior and sucrose responsiveness, respectively.

### 2.3. Orientation Behaviors

A Y maze (4 cm in diameter, 20 cm length for each arm, end of two odor arms was connected to a 600 mL glass bottle as an odor carrier) was used to determine whether bees’ orientation behaviors would be affected by a THX or THX + CAR mixture. Rape flowers were used in one arm as a test odor, while the other arm was used as a blank control. A charcoal-filtered, wet airflow was applied constantly (speed: 700 mL/min).

Returning foragers from each colony were captured. We tested how bees react to the untreated, THX-treated, THX + CAR treated flower compared with blank control. Later on, we also tested after bees consumed THX, or THX + CAR, how it will react to untreated flower, or THX-treated flower compare with blank control, or THX + CAR treated flower compared with blank control, respectively. Bees were tested one by one.

Between each of the two trials, the two arms were switched to avoid place bias. Ethanol was also used to remove the trail odors bees may leave in the tube. 30 bees from each colony were used in each treatment, and the experiments were biologically replicated three times.

The orientation behavior was divided into three different behavior categories: the first choice, duration, and the last choice in the Y maze. Therefore, three different parameters were used to assess the orientation behaviors when they encountered the non-treated flower or the chemically treated flowers. The parameters were measured as follows. If a bee entered an arm of the Y maze and proceeded into the odor area of that arm, this was counted as the first choice. Since some bees returned back and made another choice, we defined the last choice as they passed the end of the odor arm, and the duration means the time they spent from the first introduction of Y maze to their last choice.

### 2.4. Sucrose Responsiveness

Returning foragers from each colony were caught with a plastic tube, chilled on an ice block, and restrained in a small plastic tube to make sure that only their head and proboscis could move flexibly [21]. The sucrose responsiveness tests were conducted after bees were allowed to calm down after being placed in a dark and humid box for 2 h. Afterwards, bees were restrained in plastic tubes individually.

Each individual bee was first stimulated with a droplet of water and then with the following sucrose solutions (without agrochemicals) offered in ascending concentrations: 0.1, 0.3, 1, 3, 10, 30 (m/m, %) to test its proboscis extension response (PER). The inter-stimulus interval was about 4 min to exclude sugar sensitization effects [22,23]. These experiments were repeated three times, and 30 bees from each colony were used in each replicate.

### 2.5. Data Analysis

All the parameters of Y-shape maze experiment were calculated and presented as percentage towards all choices. Each choice to the reward arm was compared with the expected value of 50% with Chi-square tests. The orientation behavior among different days, among different treated bee groups and three different flower treated groups were separately analyzed by Chi-square tests.

To analyze the sugar responsiveness of bees, we separately analyzed three treatments on each day, and the same treatment method was applied on different days. Repeated measure ANOVA was used to analyze the data with treatment (Control, Insecticide (THX), Insecticide and fungicide (THX + CAR)), sucrose concentration (0–30%), and the different days as fixed effects. For post hoc testing, we used Tukey’s Honestly Significant Difference (HSD) test to do the multiple comparisons among different treatments, or among different days.

## 3. Results

### 3.1. Orientation Behaviors

**Figure 1:**
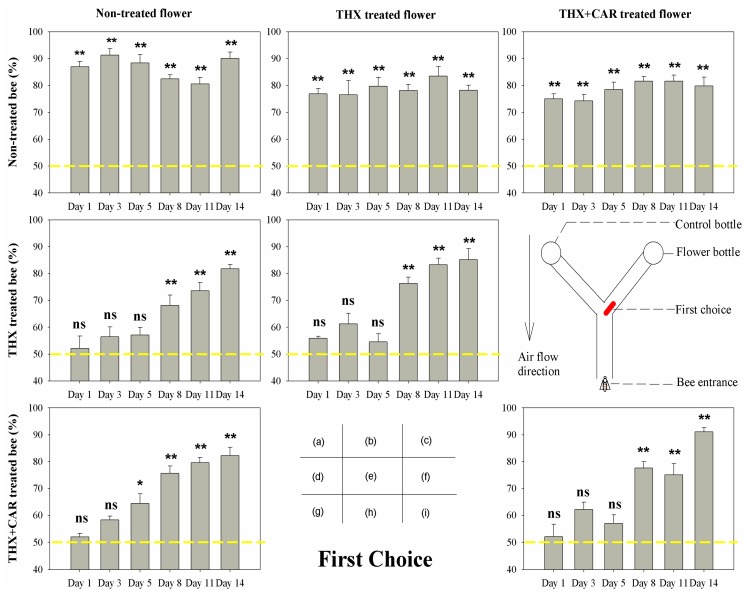
The first choice of bee orientation behavior in Y maze treated with thiamethoxam (THX) and thiamethoxam + carbendazol mixture (THX + CAR). The X-axis presented the time post-treatment (Day). The red bold line in diagram (f) showed the first choice of a bee enter to flower arm. The yellow dash line showed the reference line of 50%, * presents significant difference (*p* < 0.05), ** presents great significant difference (*p* < 0.01), ns means no significant difference (*p* > 0.05).

**Figure 2:**
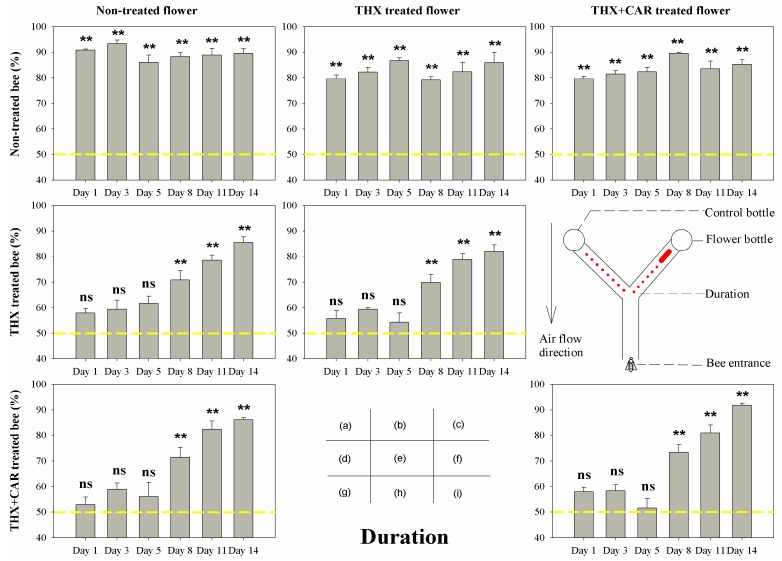
The duration of bee orientation behavior in Y maze treated with thiamethoxam (THX) and thiamethoxam + carbendazol mixture (THX + CAR). The X-axis presented the time post-treatment (Day). The red bold line and the dots in diagram (f) showed the bee wander back and forth between two arms. The yellow dash line showed the reference line of 50%, ** presents great significant difference (*p* < 0.01), ns means no significant difference (*p* > 0.05).

**Figure 3:**
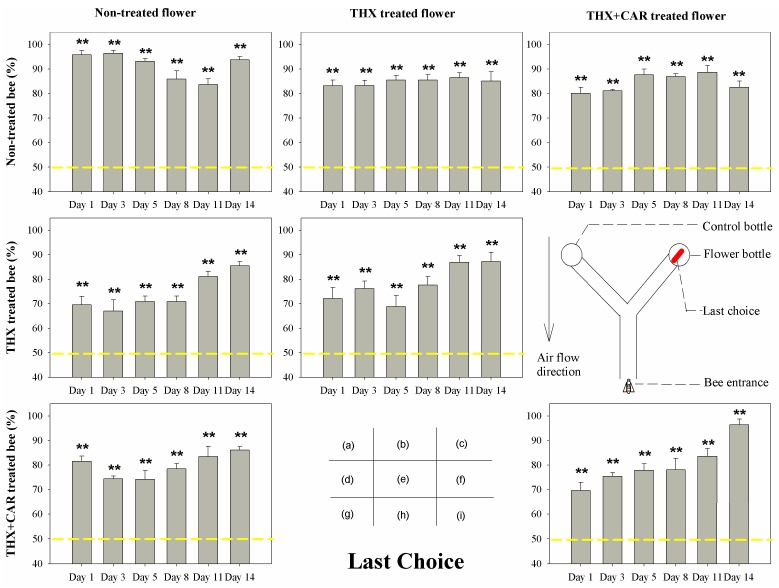
The last choice of bee orientation behavior in Y maze treated with thiamethoxam (THX) and thiamethoxam + carbendazol mixture (THX + CAR). The X-axis presented the time post-treatment (Day). The red bold line in diagram (f) showed the last choice of a bee to enter the flower bottle. The yellow dash line showed the reference line of 50%, ** presents great significant difference (*p* < 0.01), ns means no significant difference (*p* > 0.05).

The results showed that most groups would make the flower arm their first choice (Figure 1) and stay longer (Figure 2) in the flower arm than the control arm, than the control bee groups. However, in both treated groups (THX treated bee group, THX + CAR mixture treated bee group), bees could not discriminate between the blank arm and flower arm for the first choice (Figure 1) and the duration (Figure 2) was longer in the first week after the chemical treatment (1st, 3rd, and 5th day), interestingly, bees still choose the flower arm in the end (last choice, Figure 3). One week later, all groups, no matter treated or not, would chose first, stay longer at the flower arm, and choose the flower arm more than 50% of the time (Figure 1, Figure 2 and Figure 3).

Accordingly, no significant difference was found in the control group to different flower treated groups, while the chemically treated honeybees only began choosing correctly the flower arm as a first choice after they began to recover from the exposure after one week (Table 1; Figure 1, Figure 2 and Figure 3).

Bees that consumed the THX or THX + CAR mixture contaminated flower all showed signs of being mis-oriented between the flower arm and the control arm. However, in the end all bees did correctly find the flower arm in the first week. Bees would recover from this mis-orientation after one week (8th–14th day) (Table 1; Figure 1, Figure 2 and Figure 3).

When bees were not fed with insecticide nectar, they were still unable to discriminate between the non-treated flower and THX or THX + CAR mixture treated flower (Table S1). However, when bees were fed chemically treated nectar, they definitely had a longer duration in the Y maze before making the last choice during the first week after exposure (Tables S2 and S3). In the second week it may be that the THX or THX + CAR mixture residue reduced its concentrations for bees to discriminate (Tables S1–S3).

For both THX and THX + CAR mixture treated bees, there was no significant difference to discriminate between non-treated flowers or treated flowers for the first choice, duration, and last choice (Tables S1–S3).

In the first week, the control bees made the first choice right to the flower arm (no matter it was THX treated or not) more precisely than other two THX or THX + CAR mixture treated bee groups. Bees treated with THX or THX + CAR mixture were different in their first choice than the control group in that they could not tell the difference between the THX or THX + CAR mixture treated flowers (Table S4). While the bees that stayed in the Y maze were variable among the treated bee groups and the non-treated bee group in the first week (Table S5), but interestingly, bees finally did reach the flower arm, even though the non-treated bees differed in that they made it to the flower arm as the first choice more often than THX or THX + CAR mixture treated bees (Table S6).

### 3.2. Sucrose Responsiveness

**Figure 4:**
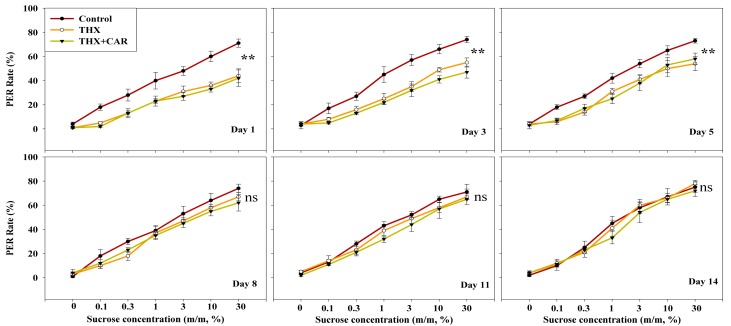
The thiamethoxam (THX) and thiamethoxam + carbendazol mixture (THX + CAR) reduced bee sugar responsiveness scores, after one week bees responded to sucrose the same as the controls. ** presents great significant difference (*p* < 0.01), ns means no significant difference (*p* > 0.05).

Bees from different greenhouses, which were treated by water, THX or THX + CAR mixture, were later tested for their sucrose responsiveness. The first day after a chemical treatment, the bees’ PER rate was reduced around a half between the control group and the chemically treated groups, no matter the treatment was THX only or the THX + CAR mixture (*F*_2,297_ = 10.925, *p* < 0.001; Figure 4). Similar effects were also found on the 3rd day (*F*_2,297_ = 9.233, *p* < 0.001) and the 5th day (*F*_2,297_ = 5.378, *p* = 0.005) after being treated with chemicals. Bees gradually recovered from the chemical treatment around the 8th day (*F*_2,297_ = 1.239, *p* = 0.291). Finally, 11 days after the chemical treatment, the chemically treated groups behaved almost the same as the control group in response to different sugar concentrations (11th Day: *F*_2,297_ = 0.973, *p* = 0.379; 14th Day: *F*_2,297_ = 0.305, *p* = 0.737, Figure 4).

There was no statistical difference found among control group bees regardless of the particular testing day (*F*_5,594_ = 0.113, *p* = 0.989). In the THX, or THX + CAR mixture, bees showed only 44–54%, 42–58% proboscis extension response gradually recovered to 78% and 72% in the 14th day (THX group: *F*_5,594_ = 4.876, *p* < 0.001; THX + CAR group: *F*_5,594_ = 4.857, *p* < 0.001, Figure 4).

## 4. Discussion

In the present study, we found that bees could not reject the flower arm even the flowers were THX or THX + CAR mixture contaminated. After bees consumed the THX or THX + CAR mixture contaminated food, bees could still find the flower reward arm in the Y maze. This result was similar to a recent study that bees prefer a sucrose solution laced with thiamethoxam, because they could not taste the difference between foods that are insecticide contaminated or not [24]. However, this was not the real case, when we looked closely into honey bee orientation behavior, we divided this behavior into three different parameters: the first choice, the duration, and the last choice. According to the first choice and the duration, the bees that consumed THX or THX + CAR mixture contaminated food, would have their ability impaired when finding out which would be the reward arm, and would wander longer in the Y maze, even though most of the bees could eventually enter the flower reward arm. This result support that the bees could not use their olfactory system to detect the scentless insecticide or fungicide over the flower odors (Figure 1, Figure 2 and Figure 3). Our sugar responsiveness experiments also showed that after the bees consumed the THX or THX + CAR mixture contaminated flowers their sugar responsiveness is reduced in comparison with the control group (Figure 4). This is supported by another group that recently published results showing an effect on sugar responsiveness after insecticide exposure [25] and indicate that scentless insecticides may not be detected by bees.

Post-consumption syndrome was visible in term of the effects on bee behavior after bees consumed the insecticide laced sucrose solution. In the present study, bees showed mis-oriented behavior mainly exhibited as a wrong first choice; however, at the final stage, most of the bees could still find the arm with the flower reward. However, during the experiment, we also found that bees (that consumed THX or THX + CAR mixture) wander back and forth from both arms to make a final decision, that cost them much more time in the Y maze than the control group. The effects on honey bee orientation are similar to most of the previous reports suggesting that thiamethoxam exposure would affect bee motor skills, and phototaxis, reducing bee health [14].

Bees need at least one week or more to recover from the insecticide post-consumption effects to behave normally. Bees in this experiment showed mis-oriented behavior mainly exhibited as a wrong first choice and using a longer duration to make the choice. Accordingly, their sugar responsiveness was also reduced by the THX or THX + CAR mixture treated food, and one week was needed for them to recover.

Both THX (0 to 663.8 ng/g) and CAR (0 to 4516.4 ng/g) residues are detected out of bee bread based on a survey close to oil rape fields in China [26]. Contamination of agrochemicals occurs because crop field treatment and the residue leaching into the soil, and the water systems [27]. In the present study, we conducted our experiments in semi-field conditions (in three nylon net covered greenhouses). The wind, sunshine, and rain could go through the net. On the 7th day there was a rain shower, which we believe washed away a lot insecticide and fungicide residue on the flowers, which resulted in bee orientation and sugar responsiveness at levels similar to the control group.

Honeybees that have been exposed to low concentrations of toxic compounds in their environment are better able to tolerate the exposure [28]. However, even at low concentrations, insecticides can potentially have devastating effects. More concerns have been raised as regards the direct effects of neonicotinoids on non-target pollinators, some countries even voting to limit or ban use of these insecticides. In the present study, our results indicate that bees are impaired by THX or THX + CAR mixture after consumption, but that they cannot detect these compounds before contact (no olfactory effects).

## 5. Conclusions

Honeybees could not discriminate scentless insecticide/fungicide contaminated flowers from the non-treated flowers, lead to post-consumption honeybees be affected by insecticide exposure on their orientation behavior and sugar responsiveness. The present study bridged the laboratory experiments and field residue surveys on insecticide induced behavioral impairments in honeybees.

## Figures and Tables

**Table 1:** The statistical results of agrochemicals effect on orientation behavior.

		First Choice		Duration		Last Choice	
		*χ^2^*	*p*	*χ^2^*	*p*	*χ^2^*	*p*
Non-treated bee	Non-treated flower	7.731	0.172	2.281	0.809	20.22	<0.001
Non-treated bee	THX treated flower	1.608	0.9	5.118	0.402	0.997	0.963
Non-treated bee	THX + CAR treated flower	3.525	0.62	4.992	0.417	5.649	0.342
THX treated bee	Non-treated flower	31.227	<0.001	27.448	<0.001	13.196	0.022
THX treated bee	THX treated flower	43.708	<0.001	32.7	<0.001	11.957	0.035
THX + CAR treated bee	Non-treated flower	34.549	<0.001	45.313	<0.001	7.49	0.187
THX + CAR treated bee	THX + CAR treated flower	46.722	<0.001	57.691	<0.001	24.39	<0.001

## Supplementary Materials

The following are available online at http://www.mdpi.com/2075-4450/9/4/130/s1, Table S1: The statistical results of agrochemicals effect on bees’ first choice to agrochemical contaminated flowers or not, Table S2: The statistical results of agrochemicals effect on bees’ last choice to agrochemical contaminated flowers or not, Table S3: The statistical results of agrochemicals effect on bees’ duration in Y maze to agrochemical contaminated flowers or not, Table S4: The statistical results of agrochemicals effect on bees’ first choice to flowers after THX, or THX+CAR consumption, Table S5: The statistical results of agrochemicals effect on bees’ duration in Y maze to flowers after THX, or THX+CAR consumption, Table S6: The statistical results of agrochemicals effect on bees’ last choice to flowers after THX, or THX+CAR consumption.

## References

[1] Potts S.G., Biesmeijer J.C., Kremen C., Neumann P., Schweiger O., Kunin W.E. (2010). Global pollinator declines: Trends, impacts and drivers. Trends Ecol. Evol..

[2] Goulson D., Nicholls E., Botías C., Rotheray E.L. (2015). Bee declines driven by combined stress from parasites, pesticides, and lack of flowers. Science.

[3] Mitchell E., Mulhauser B., Mulot M., Mutabazi A., Glauser G., Aebi A. (2017). A worldwide survey of neonicotinoids in honey. Science.

[4] Di N., Hladun K.R., Zhang K., Liu T.X., Trumble J.T. (2016). Laboratory bioassays on the impact of cadmium, copper and lead on the development and survival of honeybee (*Apis mellifera* L.) larvae and foragers. Chemosphere.

[5] Ware G.W. (1980). Effects of Pesticides on Nontarget Organisms.

[6] Hallmann C.A., Foppen R.P.B., van Turnhout C.A.M., de Kroon H., Jongejans E. (2014). Declines in insectivorous birds are associated with high neonicotinoid concentrations. Nature.

[7] Gilburn A.S., Bunnefeld N., Wilson M.V., Botham M.S., Brereton T.M., Fox R., Goulson D. (2015). Are neonicotinoid insecticides driving declines of widespread butterflies?. PeerJ.

[8] Park M.G., Blitzer E.J., Gibbs J., Losey J.E., Danforth B.N. (2015). Negative effects of pesticides on wild bee communities can be buffered by landscape context. Proc. Biol. Sci..

[9] Woodcock B.A., Isaac N.J.B., Bullock J.M., Roy D.B., Garthwaite D.G., Crowe A., Pywell R.F. (2016). Impacts of neonicotinoid use on long-term population changes in wild bees in England. Nat. Commun..

[10] Casida J.E. (2018). Neonicotinoids and Other Insect Nicotinic Receptor Competitive Modulators: Progress and Prospects. Ann. Rev. Entomol..

[11] Henry M., Beguin M., Requier F., Rollin O., Odoux J.F., Aupinel P., Aptel J., Tchamitchian S., Decourtye A. (2012). A common pesticide decreases foraging success and survival in honey bees. Science.

[12] Dai P., Jack C.J., Mortensen A.N., Bustamante T.A., Bloomquist J.R., Ellis J.D. (2018). Chronic toxicity of clothianidin, imidacloprid, chlorpyrifos, and dimethoate to *Apis mellifera* L. larvae reared in vitro. Pest Manag. Sci..

[13] Tosi S., Burgio G., Nieh J.C. (2017). A common neonicotinoid pesticide, thiamethoxam, impairs honey bee flight ability. Sci. Rep..

[14] Tosi S., Nieh J.C. (2017). A common neonicotinoid pesticide, thiamethoxam, alters honey bee activity, motor unctions, and movement to light. Sci. Rep..

[15] Gill R.J., Ramosrodriguez O., Raine N.E. (2012). Combined pesticide exposure severely affects individual- and colony-level traits in bees. Nature.

[16] Tsvetkov N., Samson-Robert O., Sood K., Patel H.S., Malena D.A., Gajiwala P.H., Maciukiewicz P., Fournier V., Zayed A. (2017). Chronic exposure to neonicotinoids reduces honey bee health near corn crops. Science.

[17] Schaafsma A., Limayrios V., Baute T., Smith J., Xue Y. (2015). Neonicotinoid insecticide residues in surface water and soil associated with commercial maize (corn) fields in southwestern Ontario. PLoS ONE.

[18] Botías C., David A., Hill E.M., Goulson D. (2016). Contamination of wild plants near neonicotinoid seed-treated crops, and implications for non-target insects. Sci. Total Environ..

[19] Oliveira R.A., Roat T.C., Carvalho S.M., Malaspina O. (2014). Side-effects of thiamethoxam on the brain andmidgut of the africanized honeybee *Apis mellifera* (Hymenopptera: Apidae). Environ. Toxicol..

[20] Wang K., Pang Q., Zhang W.W., Ting J.I. (2017). Effects of sublethal doses of carbendazim on the growth and detoxifying enzyme activities of honeybee(*Apis mellifera ligustica*) larvae. Acta Entomol. Sin..

[21] Wang Z., Qu Y., Dong S., Wen P., Li J., Tan K., Menzel R. (2016). Honey bees modulate their olfactory learning in the presence of hornet predators and alarm component. PLoS ONE.

[22] Scheiner R., Kuritz-Kaiser A., Menzel R., Erber J. (2005). Sensory responsiveness and the effects of equal subjective rewards on tactile learning and memory of honeybees. Learn. Mem..

[23] Wang Z., Tan K. (2014). Comparative analysis of olfactory learning of *Apis cerana* and *Apis mellifera*. Apidologie.

[24] Kessler S.C., Tiedeken E.J., Simcock K.L., Derveau S., Mitchell J., Softley S., Stout J.C., Wright G.A. (2015). Bees prefer foods containing neonicotinoid pesticides. Nature.

[25] Démares F.J., Pirk C.W.W., Nicolson S.W., Human H. (2018). Neonicotinoids decrease sucrose responsiveness of honey bees at first contact. J. Insect Physiol..

[26] Tong Z., Duan J., Wu Y., Liu Q., He Q., Shi Y., Yu L., Cao H. (2018). A survey of multiple pesticide residues in pollen and beebread collected in China. Sci. Total Environ..

[27] Székács A., Mörtl M., Darvas B. (2015). Monitoring pesticide residues in surface and ground water in Hungary: Surveys in 1990–2015. J. Chem..

[28] Johnson R.M. (2015). Honey bee toxicology. Annu. Rev. Entomol..

