# Supplementary material for: The Effect of Neonicotinoid Insecticide and Fungicide on Sugar Responsiveness and Orientation Behavior of Honey Bee (Apis mellifera) in Semi-Field Conditions
Source: Insects. 2018 Sep 29;9(4):130. doi: 10.3390/insects9040130 (PMC6316467; doi:10.3390/insects9040130)
Supplement: Supplementary file 1 [file insects-09-00130-s001.pdf]

Table S1. The statistical results of agrochemicals effect on bees' first choice to agrochemical contaminated flowers or not.

**First choice**		day1		day3		day5		day8		day11		day14	
		χ^2	p	χ^2	p	χ^2	p	χ^2	p	χ^2	p	χ^2	p
Non-treated bee	Non-treated flower												
Non-treated bee	THX treated flower	5.103	0.078	10.559	0.005	4.721	0.094	0.684	0.71	0.136	0.934	5.769	0.056
Non-treated bee	THX+CAR treated flower												
THX treated bee	Non-treated flower												
THX treated bee	THX treated flower	0.322	0.57	0.515	0.473	0.081	0.776	0.913	0.339	2.4	0.121	0.327	0.568
THX+CAR treated bee	Non-treated flower												
THX+CAR treated bee	THX+CAR treated flower	0.181	0.671	0.333	0.564	1.345	0.246	0.113	0.737	0.452	0.502	3.468	0.063

Table S2. The statistical results of agrochemicals effect on bees' last choice to agrochemical contaminated flowers or not.

**Last choice**		day1		day3		day5		day8		day11		day14	
		χ^2	p	χ^2	p	χ^2	p	χ^2	p	χ^2	p	χ^2	p
Non-treated bee	Non-treated flower												
Non-treated bee	THX treated flower												
Non-treated bee	THX+CAR treated flower	12.261	0.002	11.481	0.003	3.251	0.197	0.166	0.92	1.582	0.453	6.207	0.045
THX treated bee	Non-treated flower												
THX treated bee	THX treated flower	0.097	0.755	1.987	0.159	0.095	0.758	1.29	0.256	1.339	0.247	0.385	0.535
THX+CAR treated bee	Non-treated flower												
THX+CAR treated bee	THX+CAR treated flower	3.365	0.067	0.026	0.871	0.439	0.508	0.03	0.863	0.036	0.849	6.105	0.13

Table S3. The statistical results of agrochemicals effect on bees' duration in Y maze to agrochemical contaminated flowers or not.

**Duration**		day1		day3		day5		day8		day11		day14	
		χ^2	p	χ^2	p	χ^2	p	χ^2	p	χ^2	p	χ^2	p
Non-treated bee	Non-treated flower												
Non-treated bee	THX treated flower												
Non-treated bee	THX+CAR treated flower	6.945	0.031	7.085	0.029	1.332	0.514	6.146	0.046	2.610	0.271	0.707	0.702
THX treated bee	Non-treated flower												
THX treated bee	THX treated flower	0.082	0.775	0	1	1.314	0.252	0	1	0	1	0.149	0.7
THX+CAR treated bee	Non-treated flower												
THX+CAR treated bee	THX+CAR treated flower	0.506	0.477	0	1	0.502	0.478	0.025	0.874	0.033	0.856	1.839	0.175

Table S4. The statistical results of agrochemicals effect on bees' first choice to flowers after THX, or THX+CAR consumption.

**First choice**		day1		day3		day5		day8		day11		day14	
		χ^2	p	χ^2	p	χ^2	p	χ^2	p	χ^2	p	χ^2	p
Non-treated bee	Non-treated flower												
THX treated bee	Non-treated flower												
THX+CAR treated bee	Non-treated flower	58.080	<0.001	48.381	<0.001	35.196	<0.001	6.437	0.04	2.432	0.298	7.973	0.019
Non-treated bee	THX treated flower												
THX treated bee	THX treated flower	9.898	0.002	9.898	0.002	14.345	<0.001	0.113	0.737	0	1	1.625	0.202
Non-treated bee	THX+CAR treated flower												
THX+CAR treated bee	THX+CAR treated flower	8.791	0.003	3.309	0.069	10.051	0.002	0.5	0.48	1.452	0.228	4.88	0.027

Table S5. The statistical results of agrochemicals effect on bees' duration in Y maze to flowers after THX, or THX+CAR consumption.

**Duration**		day1		day3		day5		day8		day11		day14	
		χ^2	p	χ^2	p	χ^2	p	χ^2	p	χ^2	p	χ^2	p
Non-treated bee	Non-treated flower												
THX treated bee	Non-treated flower												
THX+CAR treated bee	Non-treated flower	38.765	<0.001	36.117	<0.001	24.986	<0.001	12.309	0.002	5.376	0.068	0.750	0.687
Non-treated bee	THX treated flower												
THX treated bee	THX treated flower	10.945	0.001	11.753	0.001	26.181	<0.001	2.132	0.144	0.796	0.372	0.627	0.428
Non-treated bee	THX+CAR treated flower												
THX+CAR treated bee	THX+CAR treated flower	11.314	0.001	11.524	0.001	21.569	<0.001	9.584	0.002	0.136	0.713	2.407	0.121

Table S6. The statistical results of agrochemicals effect on bees' last choice to flowers after THX, or THX+CAR consumption.

**Last choice**		day1		day3		day5		day8		day11		day14	
		χ^2	p	χ^2	p	χ^2	p	χ^2	p	χ^2	p	χ^2	p
Non-treated bee	Non-treated flower												
THX treated bee	Non-treated flower												
THX+CAR treated bee	Non-treated flower	23.635	<0.001	31.342	<0.001	17.362	<0.001	5.813	0.055	0.183	0.912	4.722	0.094
Non-treated bee	THX treated flower												
THX treated bee	THX treated flower	3.47	0.063	1.503	0.22	7.228	0.007	2.168	0.141	0	1	0.385	0.535
Non-treated bee	THX+CAR treated flower												
THX+CAR treated bee	THX+CAR treated flower	2.189	0.139	1.049	0.306	3.544	0.06	2.805	0.094	1.07	0.301	8.992	0.003
